# Supplementary figures and images for: Murine alveolar macrophages rapidly accumulate intranasally administered SARS-CoV-2 Spike protein leading to neutrophil recruitment and damage
Source: eLife. 2024 Mar 20;12:RP86764. doi: 10.7554/eLife.86764 (PMC10954308; doi:10.7554/eLife.86764)

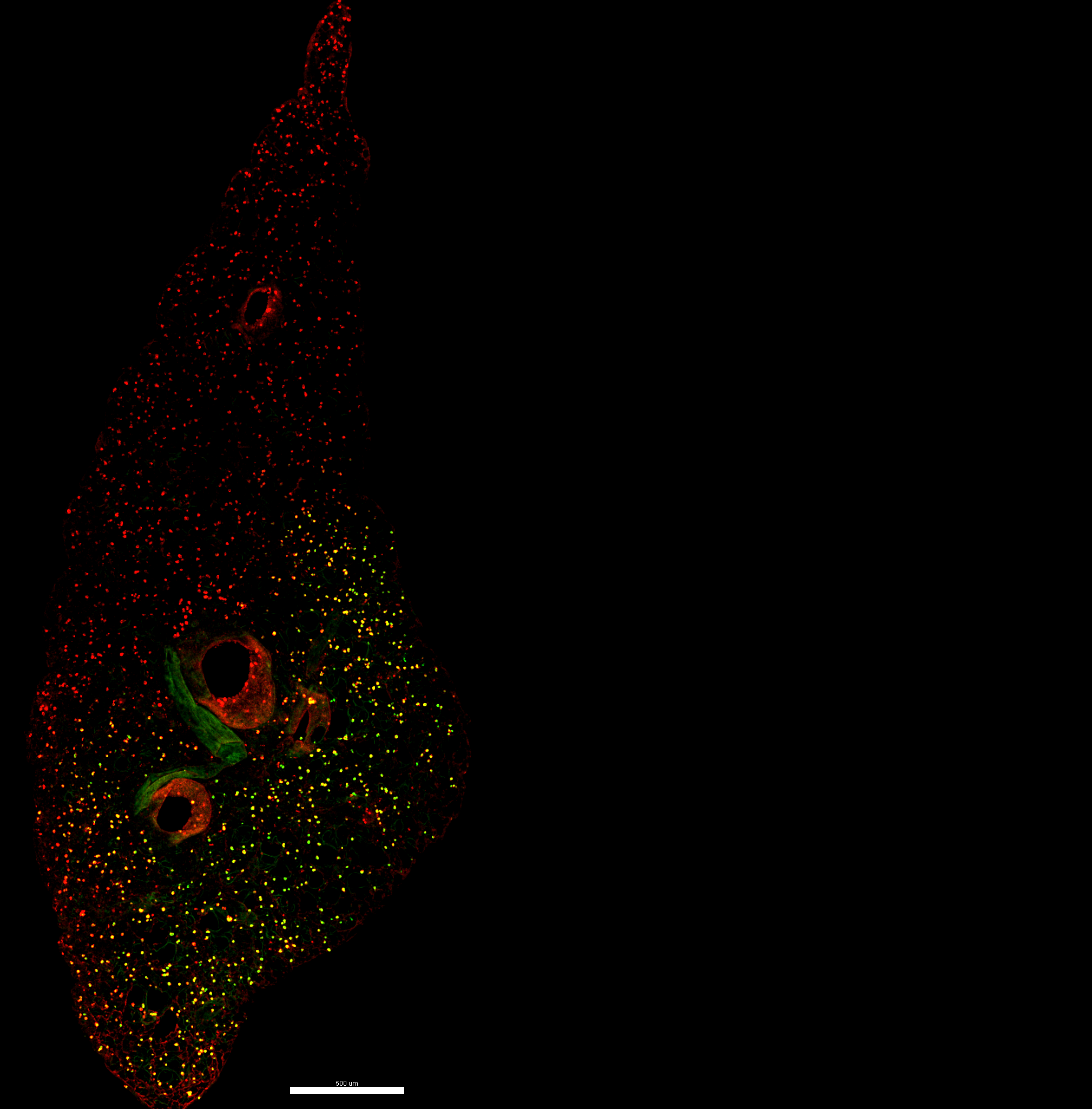

Supplement: Figure 1—source data 1. [file elife-86764-fig1-data1.zip › Figure 1A.tif]

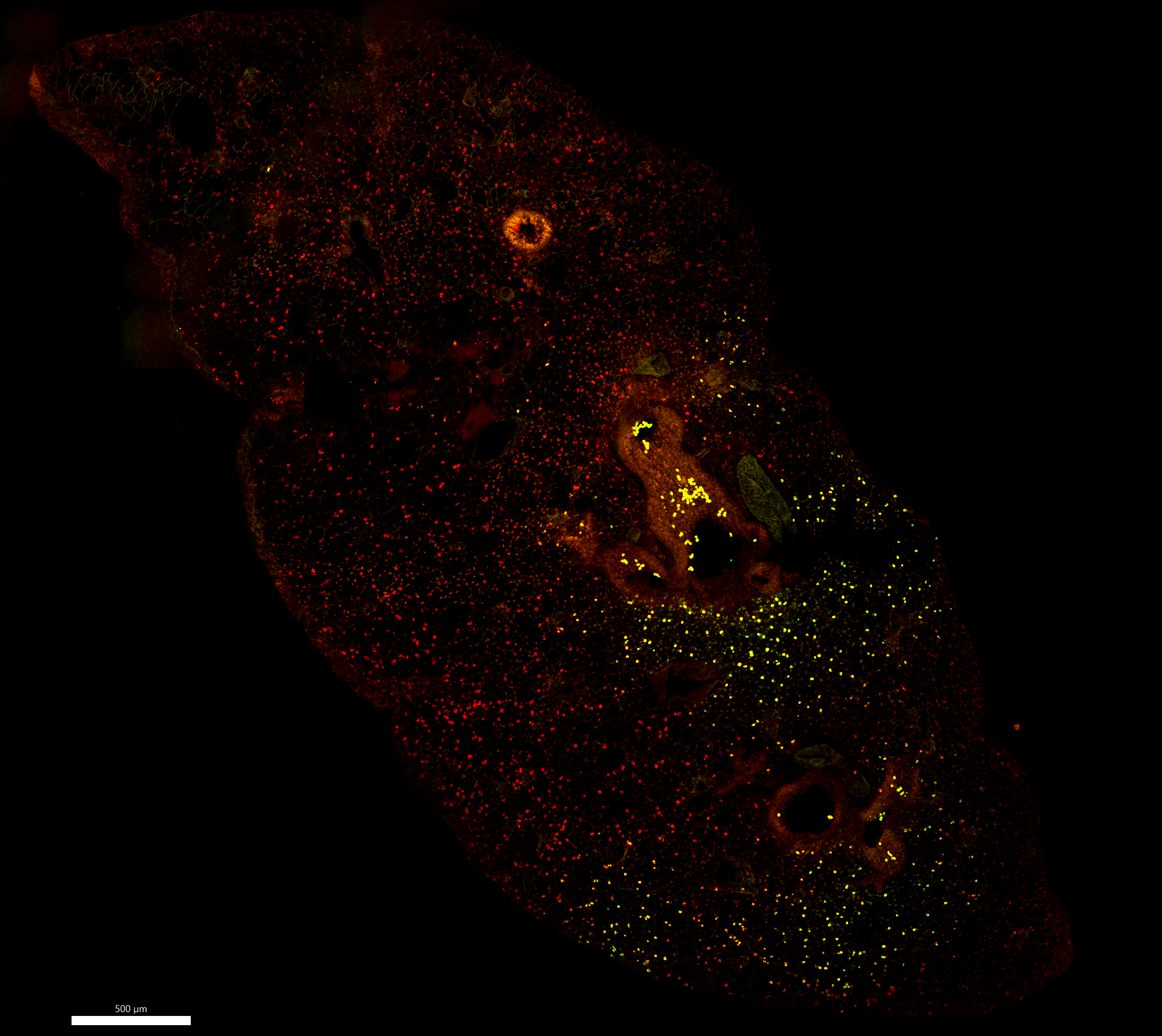

Supplement: Figure 1—source data 2. [file elife-86764-fig1-data2.zip › Figure 1B upper left.tif]

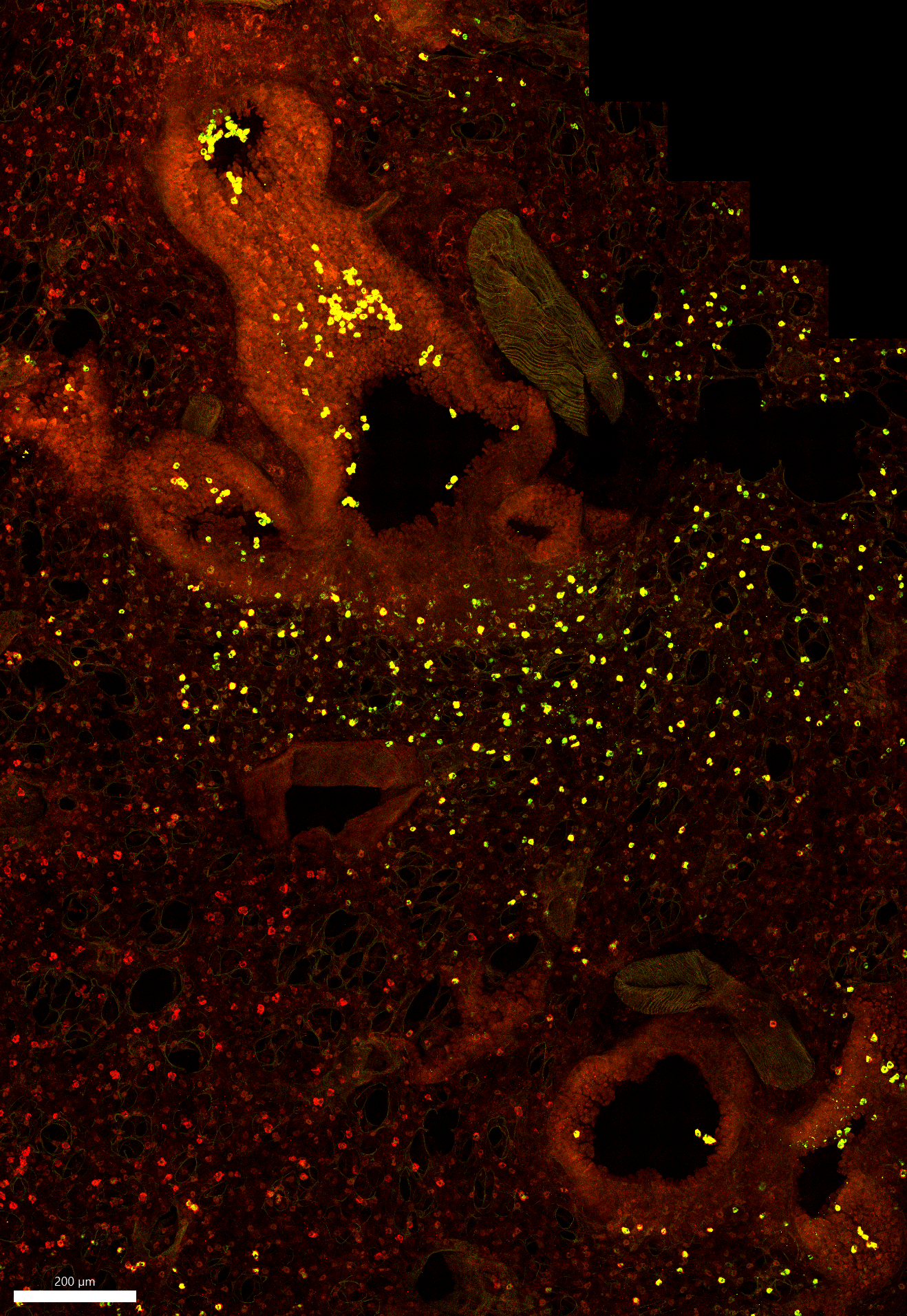

Supplement: Figure 1—source data 3. [file elife-86764-fig1-data3.zip › Figure 1B upper right.tif]

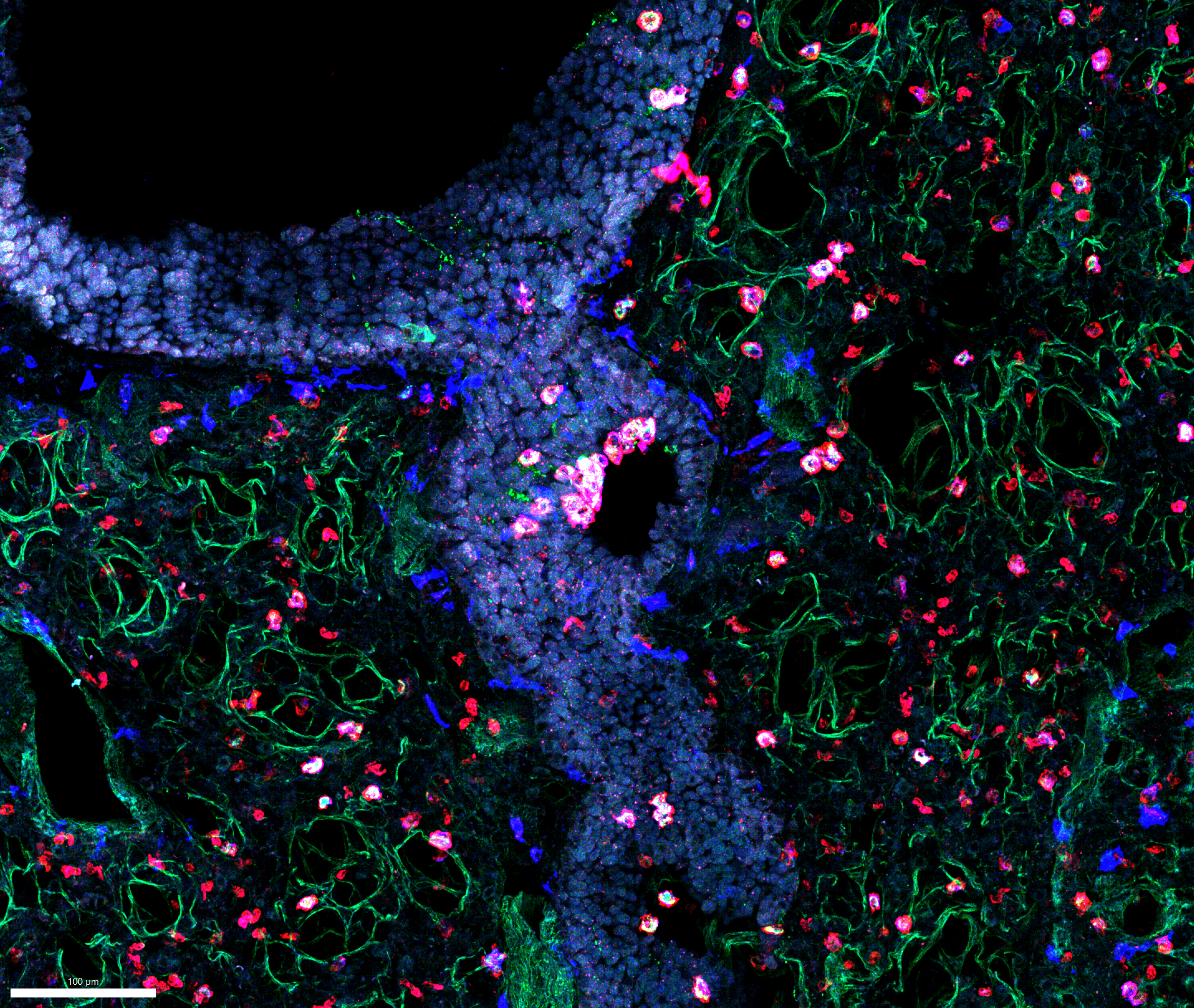

Supplement: Figure 1—source data 4. [file elife-86764-fig1-data4.zip › Figure 1D.tif]

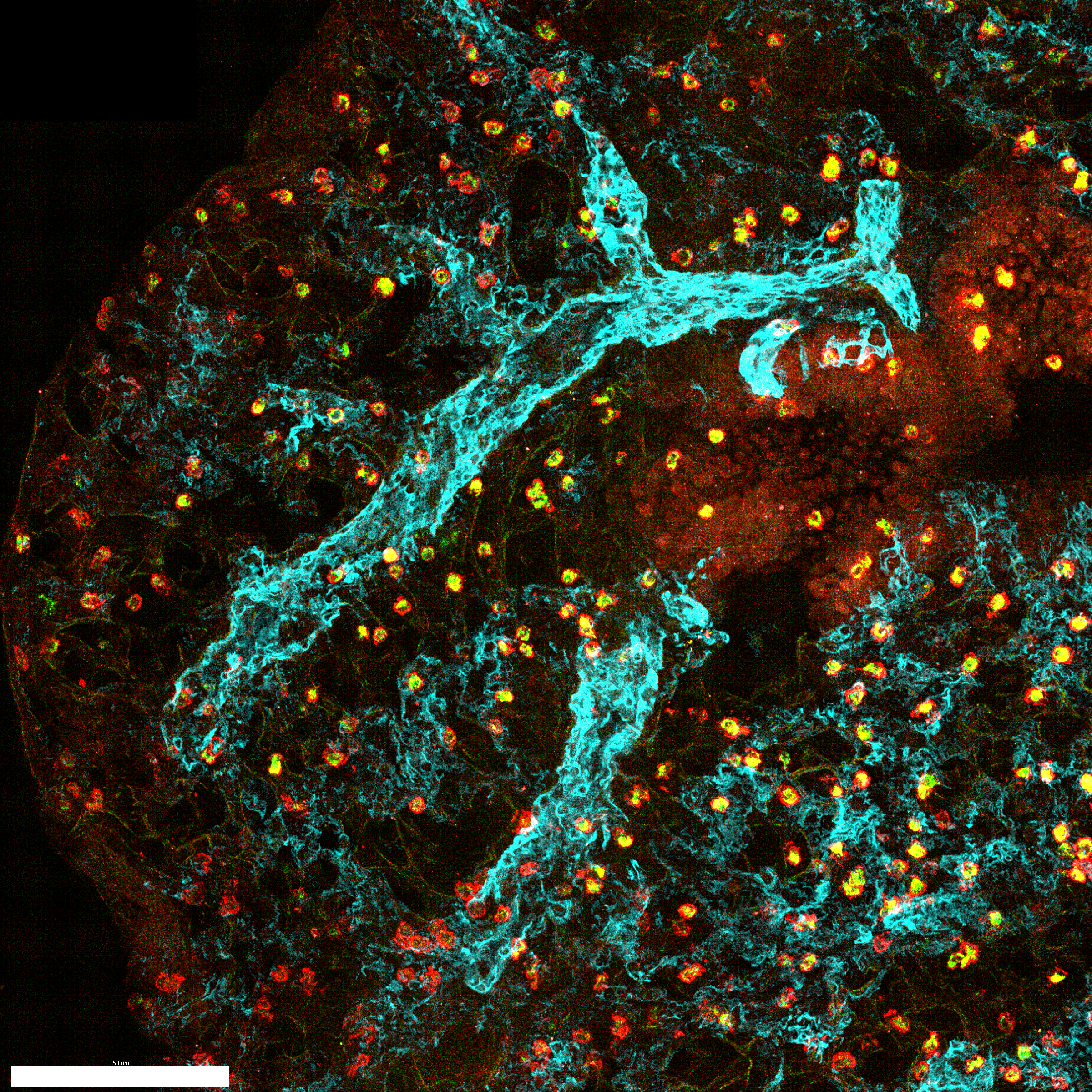

Supplement: Figure 1—source data 5. [file elife-86764-fig1-data5.zip › Figure 1G.tif]

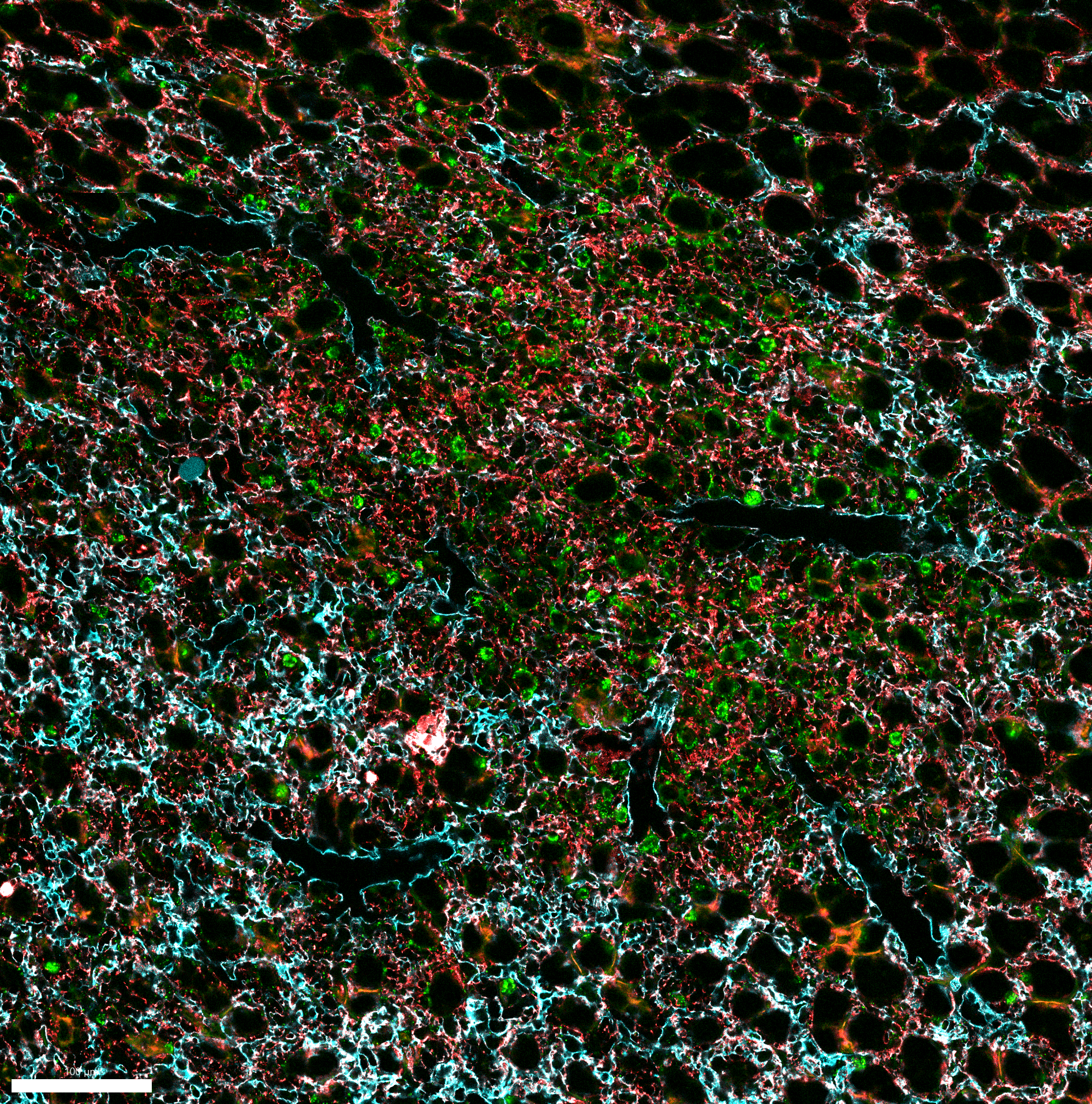

Supplement: Figure 1—source data 6. [file elife-86764-fig1-data6.zip › Figure 1I.tif]

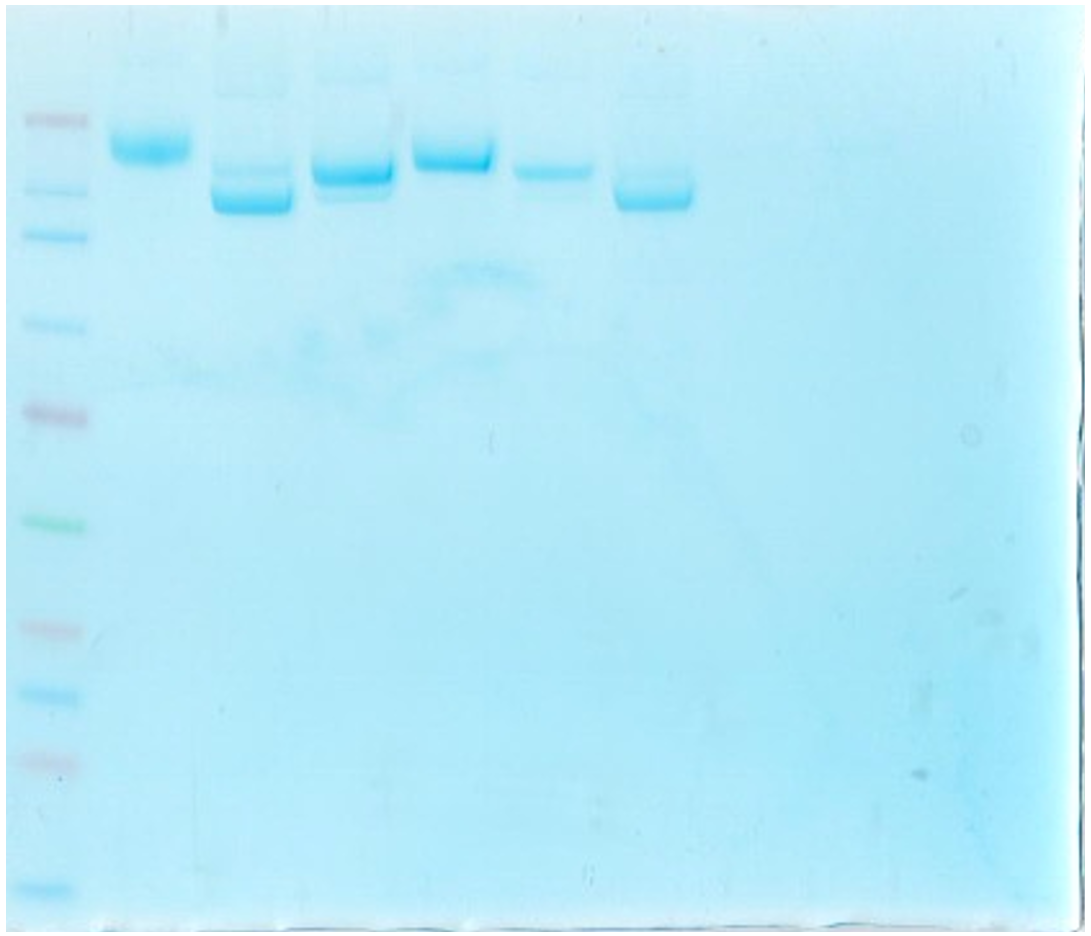

Supplement: Figure 1—figure supplement 1—source data 1. [file elife-86764-fig1-figsupp1-data1.tif]

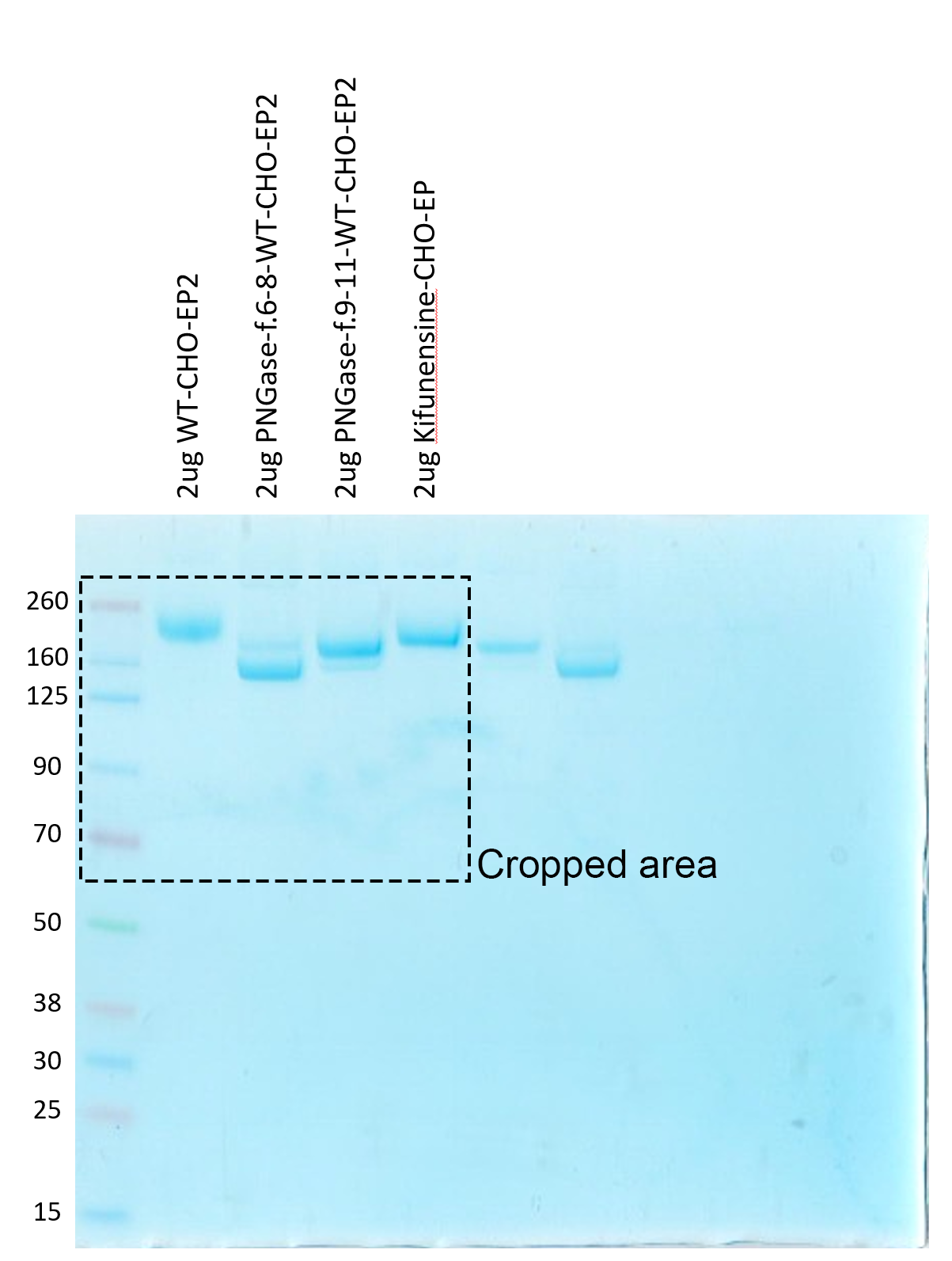

Supplement: Figure 1—figure supplement 1—source data 2. [file elife-86764-fig1-figsupp1-data2.tif]

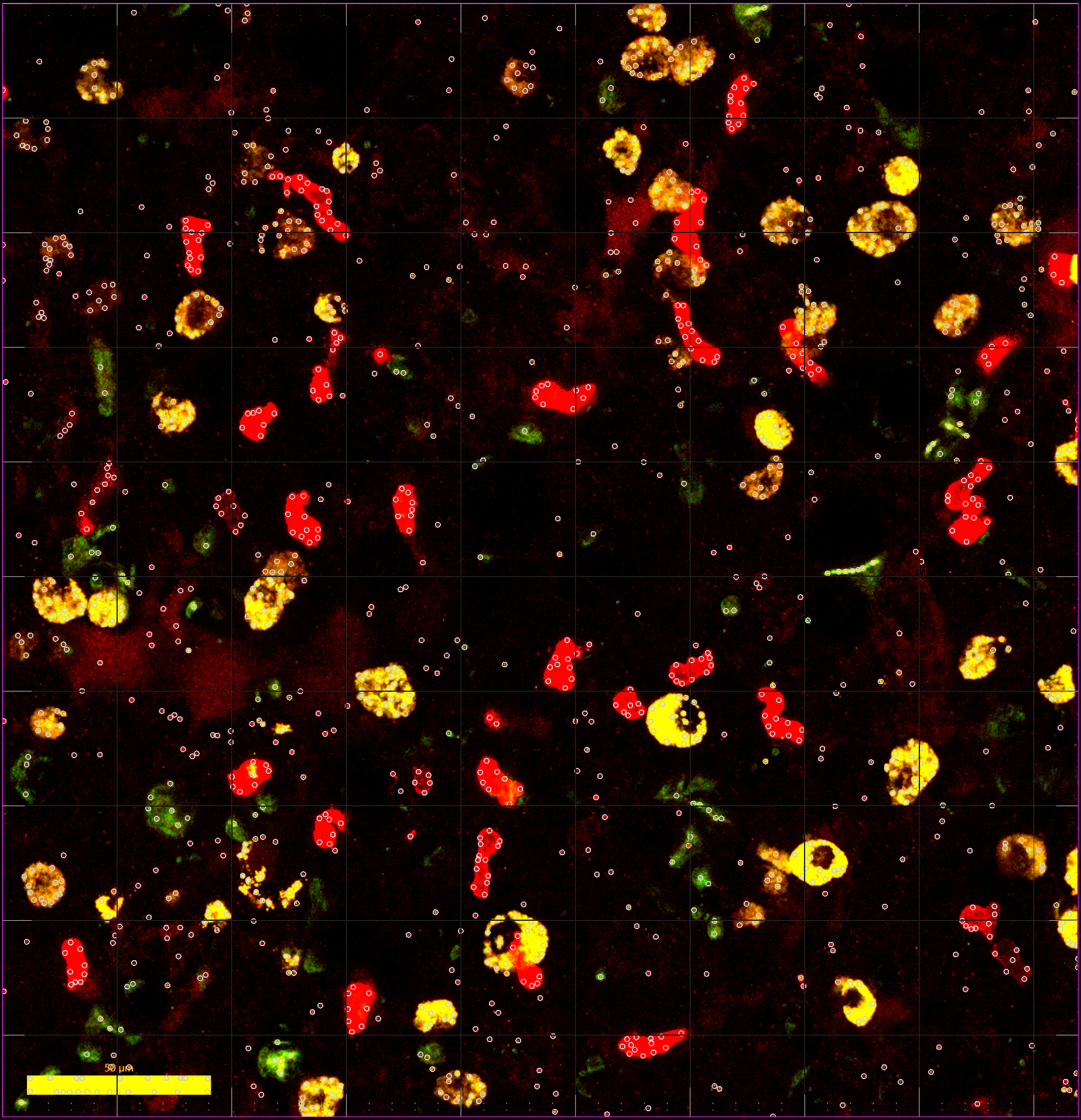

Supplement: Figure 1—figure supplement 2—source data 2. [file elife-86764-fig1-figsupp2-data2.zip › Figure 1-figure supplement 2 dEnv VLP.tif]

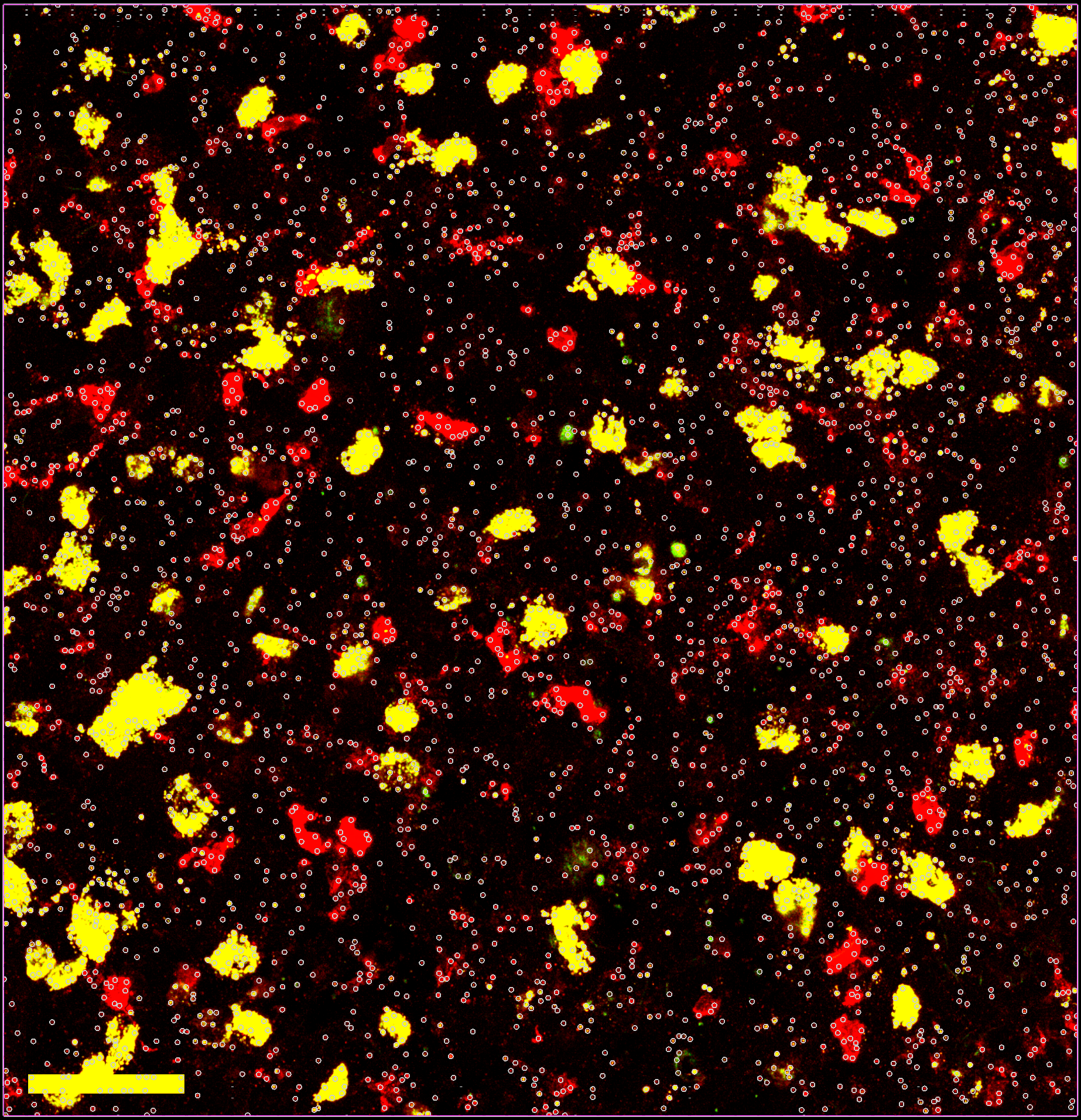

Supplement: Figure 1—figure supplement 2—source data 3. [file elife-86764-fig1-figsupp2-data3.zip › Figure 1-figure supplement 2 Spk VLP.tif]

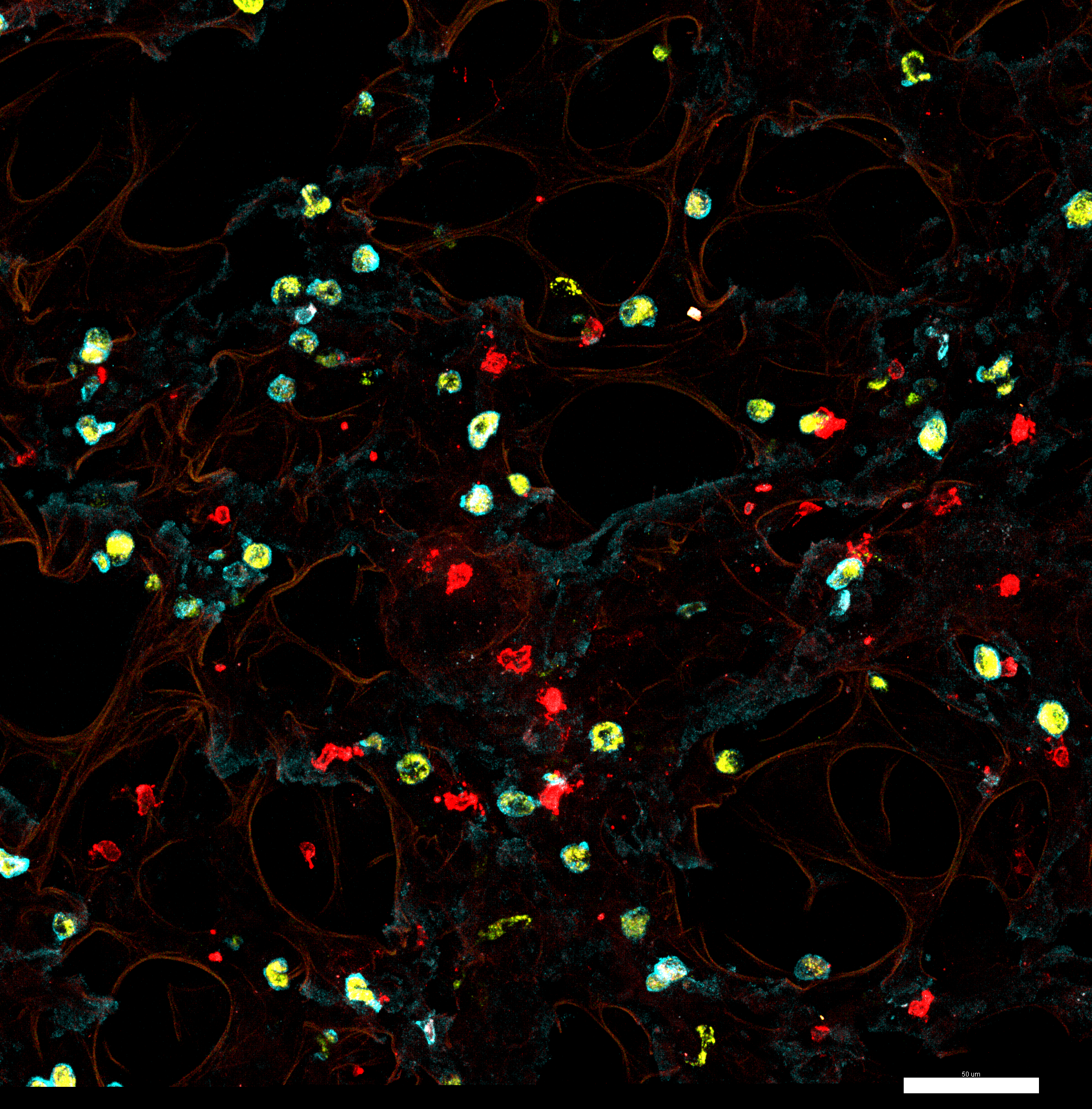

Supplement: Figure 3—source data 2. [file elife-86764-fig3-data2.zip › Figure 3C.tif]

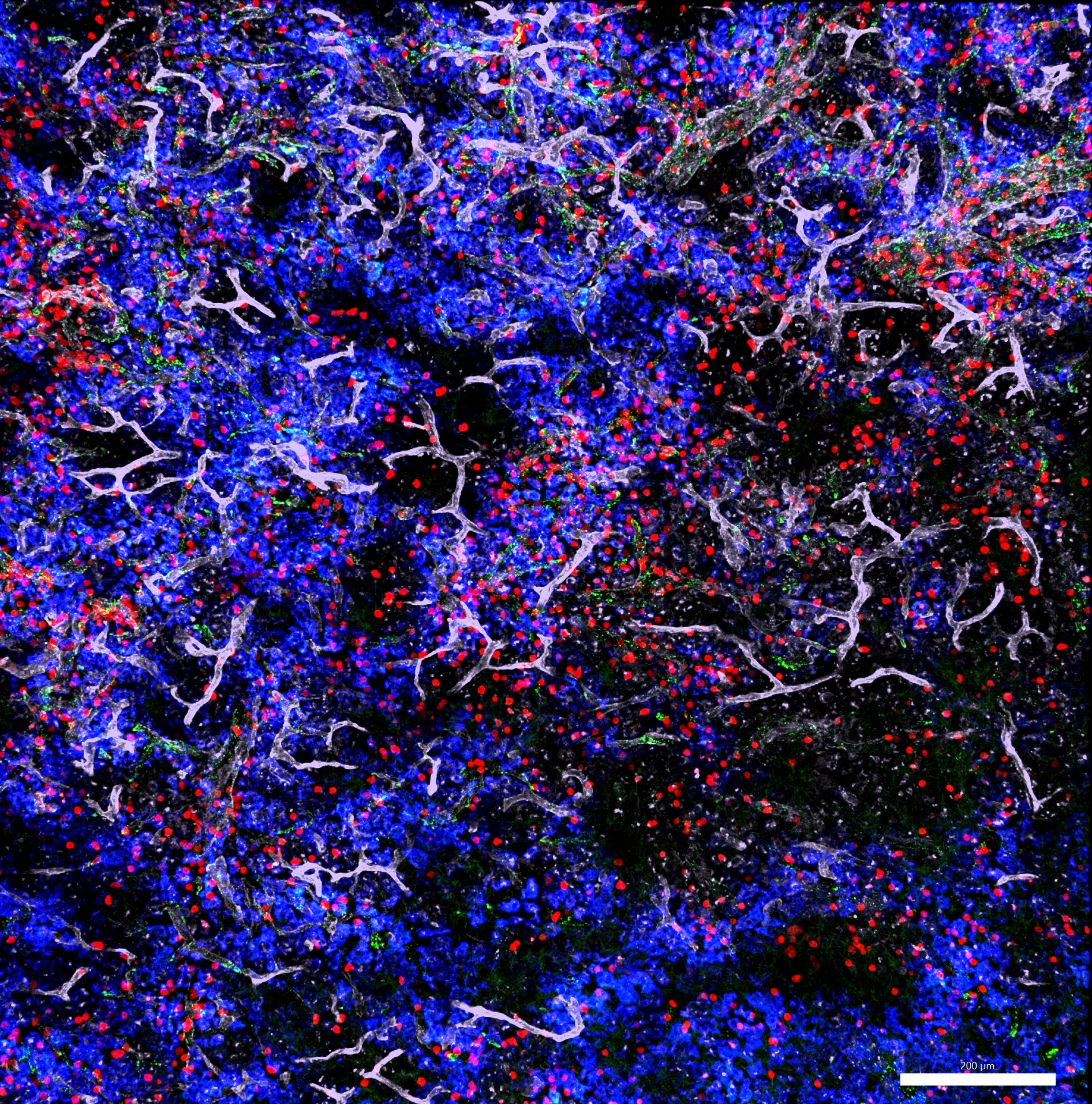

Supplement: Figure 4—figure supplement 3—source code 1. [file elife-86764-fig4-figsupp3-code1.zip › Figure 4-figure supplement 3A.tif]

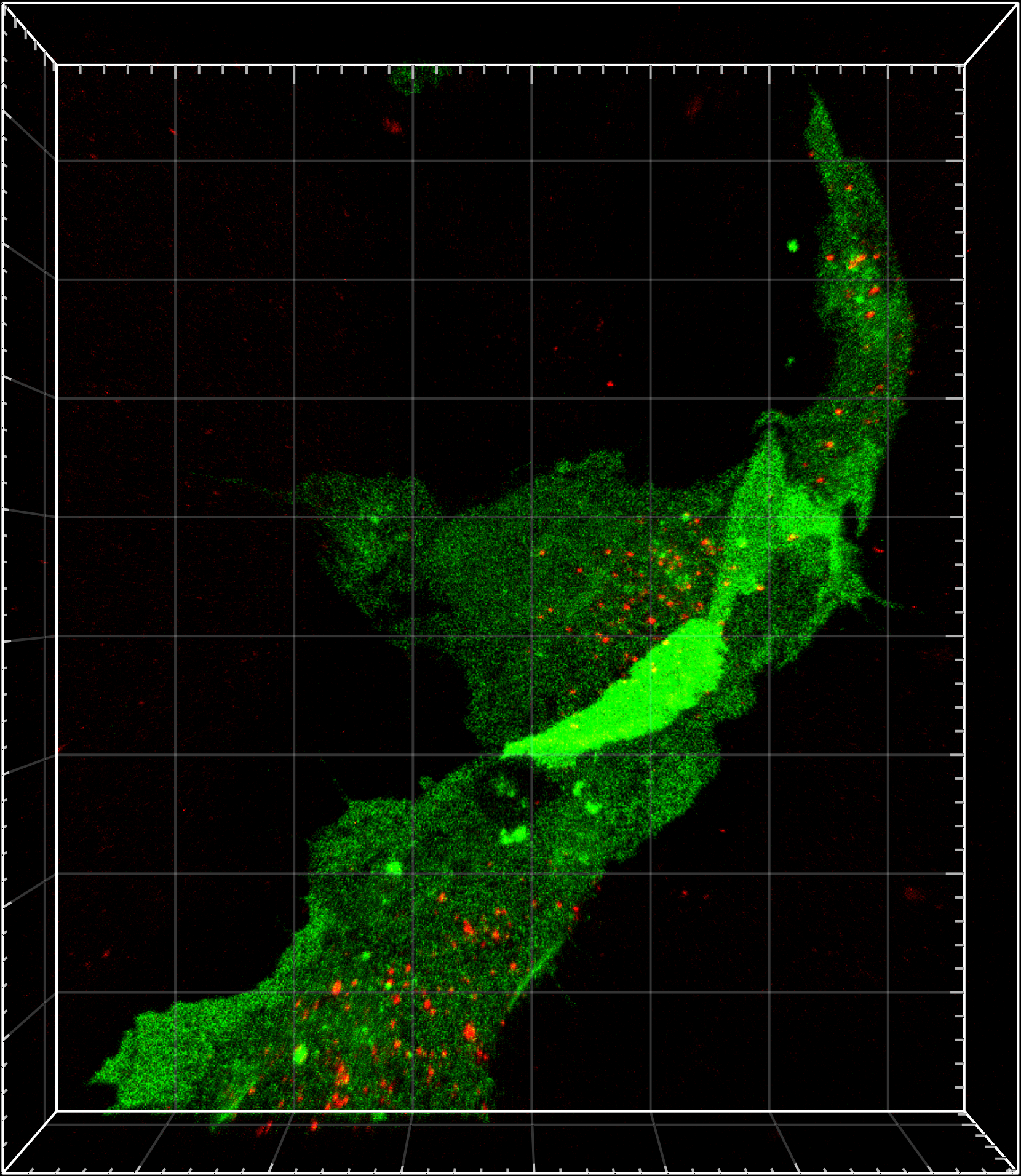

Supplement: Figure 7—source data 4. [file elife-86764-fig7-data4.zip › Figure 7D 3D reconstitution 1.tif]
